# Hypokalemia-Induced Rhabdomyolysis Secondary to Adrenal Adenoma: A Case Report

**DOI:** 10.7759/cureus.75063

**Published:** 2024-12-03

**Authors:** Faateh A Rauf, Zainab Pervaiz, Taleah Khan, Gowri Swaminathan, Theo Trandafirescu

**Affiliations:** 1 Internal Medicine, Icahn School of Medicine at Mount Sinai/New York City Health + Hospitals - Queens, New York City, USA; 2 Internal Medicine, Combined Military Hospital (CMH) Lahore Medical College and Institute of Dentistry, Lahore, PAK; 3 Pediatrics, Combined Military Hospital (CMH) Lahore Medical College and Institute of Dentistry, Lahore, PAK; 4 Pulmonary and Critical Care Medicine, Icahn School of Medicine at Mount Sinai/New York City Health + Hospitals - Queens, New York City, USA

**Keywords:** adrenal adenoma, conn's syndrome, hypokalemia, hypokalemia induced rhabdomyolysis, non-traumatic rhabdomyolysis

## Abstract

Adrenal adenoma, which leads to increased production of the hormone aldosterone, commonly presents as hypertension and hypokalemia. Rhabdomyolysis as a result of hypokalemia secondary to primary hyperaldosteronism is a rare but important complication with only a few reported cases. Low potassium levels can disrupt the regulation of arteriolar musculature, leading to reduced blood flow to skeletal muscles. This hypoperfusion may ultimately result in ischemia and cause rhabdomyolysis. We present the case of a woman with complaints of weakness and fatigue; laboratory reports showed hypokalemia and elevated serum creatine kinase (CK), leading to a diagnosis of hypokalemia-induced rhabdomyolysis. Further investigation revealed an adrenal adenoma, causing elevated aldosterone levels, which was then treated with a laparoscopic adrenalectomy, leading to the resolution of her symptoms.

## Introduction

Hypokalemia-induced rhabdomyolysis caused by primary hyperaldosteronism is a rare occurrence. Primary hyperaldosteronism, also known as Conn's syndrome, is typically due to bilateral adrenal hyperplasia (BAH) or, less commonly, an adrenal adenoma. Hyperaldosteronism leads to sodium retention and potassium excretion. It mostly presents as hypertension, although a minority of patients may experience symptoms of hypokalemia, such as muscle weakness, cramps, and fatigue [1]. A severe decrease in serum potassium levels can disrupt the skeletal muscle vascular dilation system, which results in ischemia and necrosis of the muscle fibers, leading to rhabdomyolysis. Rhabdomyolysis classically manifests as progressive weakness, fatigue, and dark urine due to the release of myoglobin and other toxic intracellular elements such as creatine kinase (CK) and lactic acid. Myoglobin has the potential to cause acute kidney injury (AKI), and in severe cases, rhabdomyolysis can also be fatal. More common causes of rhabdomyolysis include traumatic muscle injuries, exertion due to exercise or seizures, and alcohol abuse [2]. In this case, the patient used a massage gun to relieve the soreness in her legs, the use of which has been reported to result in rhabdomyolysis [3]. However, further investigation revealed a left-sided adrenal adenoma, which, after being surgically removed, led to the resolution of her symptoms, suggesting that the causative factor in this case was hyperaldosteronism, leading to hypokalemia.

## Case presentation

We present the case of a 37-year-old female with a prior history of hypertension, diabetes, and schizoaffective disorder. She was also a known case of chronic hypokalemia secondary to primary hyperaldosteronism. She presented to the Emergency Department (ED) with complaints of bilateral lower extremity weakness for seven days, along with fatigue. She had been using a massage gun extensively to alleviate the discomfort in her legs. A day before her presentation, she experienced extreme weakness and was unable to hold up her weight and fell on her lower back. She denied other symptoms like fever, dizziness, abdominal pain, cough, shortness of breath, or headache. However, a few nights ago, she experienced chest discomfort without pain and was unable to fall asleep.

A year ago, during her pregnancy, she had multiple presentations with similar symptoms of weakness. Her lab results showed raised aldosterone levels (113 ng/dL, reference values < 3.0-23.2 ng/dL), decreased plasma renin levels (0.59 pg/mL/hour, reference values ≤33.2 pg/mL/hour), and decreased serum potassium levels (3.0 mmol/L, reference values 3.5-5.1 mmol/L), for which she received both IV and oral potassium chloride (KCl). She was discharged with a prescription for KCl supplements, which she had run out of a week prior to her ED visit. She had not obtained a refill due to her upcoming appointment with an endocrinologist. 

**Figure 1:**
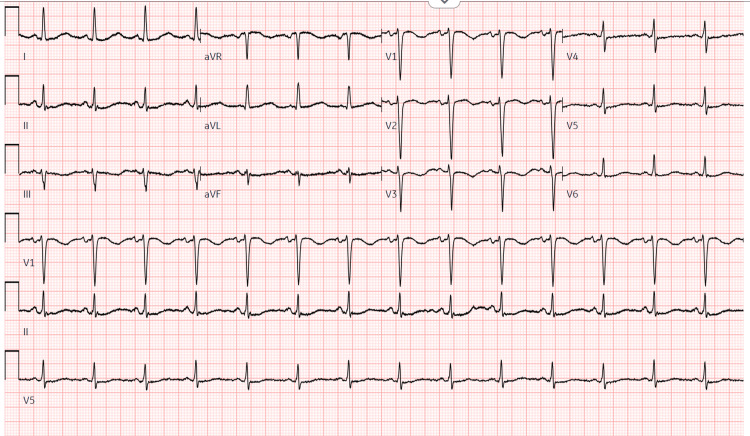
EKG showing U waves on presentation

**Figure 2:**
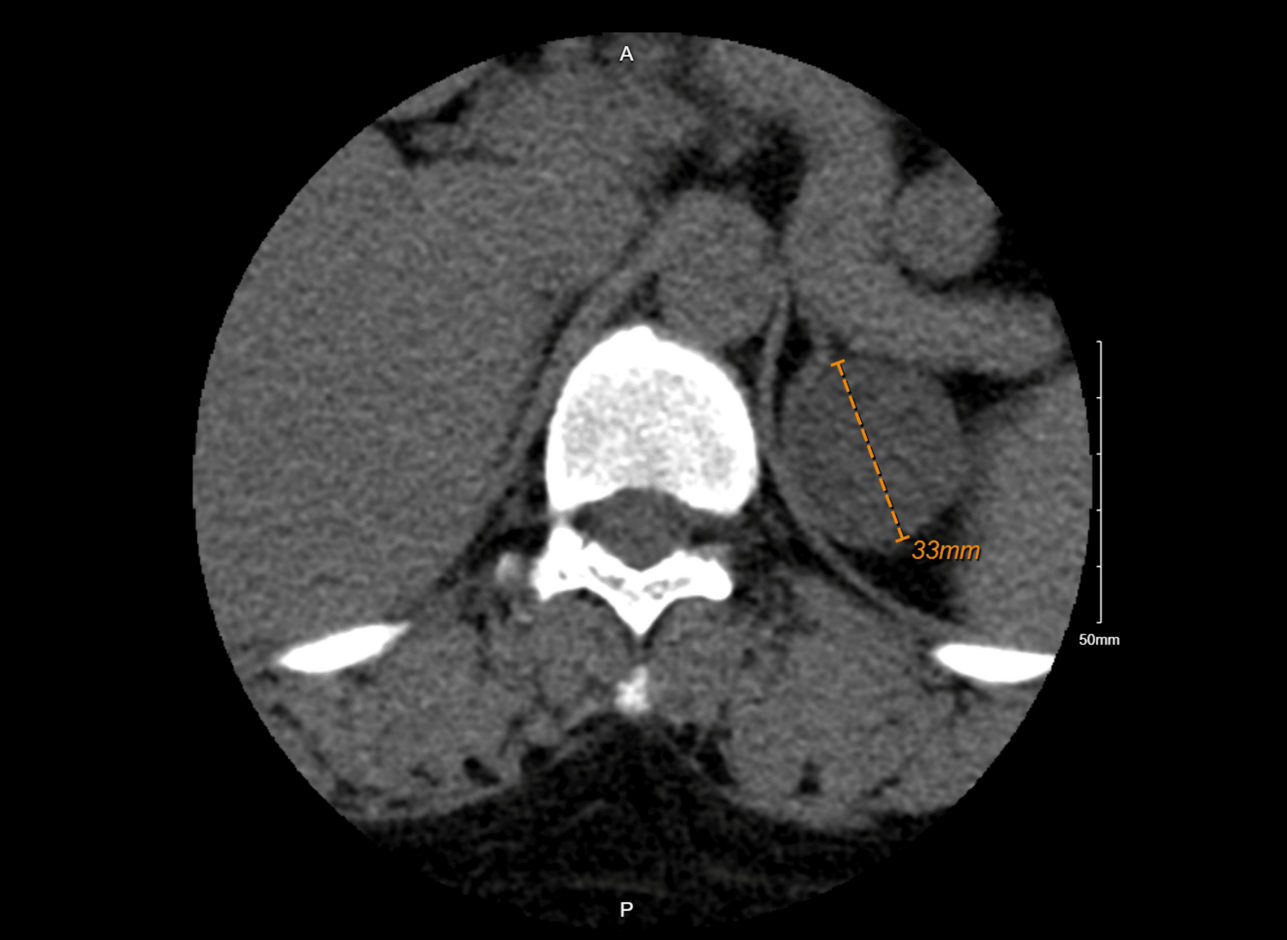
CT lumbar spine showing a 3.3 cm low-density cystic mass arising from the left adrenal gland versus the superior pole of the left kidney

Blood work at her current presentation showed an initial serum potassium level of 2.6 mmol/L and a raised CK level (9,306 U/L, reference values 20-170 U/L). Blood pressure was 153/80 mmHg. EKG showed U waves (Figure 1). A computed tomography (CT) lumbar spine scan was done in the ED to evaluate the bilateral lower extremity weakness, which revealed a 3.3 cm low-density cystic mass arising from the left adrenal gland versus the superior pole of the left kidney (Figure 2). She received 3 L of normal saline (NS) in the ED and KCl three times. Her potassium level dropped to 1.9 mmol/L, and she received IV KCl twice and 80 mEq orally; however, her potassium further dropped to 1.7 mmol/L, and she was admitted to the stepdown unit (SDU).

In the SDU, endocrinology was consulted, and the patient was started on D5-half NS with 40 mEq KCl and 100 mg spironolactone. Serum potassium remained low despite aggressive repletion, and CK levels continued to rise despite aggressive hydration and no further massage gun usage. CK levels rose to a maximum of 22,000 U/L and began to downtrend when potassium started to normalize in a sustained manner (Table 1 and Table 2). Her erythrocyte sedimentation rate (ESR) and C-reactive protein (CRP) were minimally elevated, ruling out inflammatory myopathy. The patient was discharged once stable and had a follow-up with an endocrinologist and endocrine surgeon. 

**Table 1:** Sodium and potassium trends during the course of admission

	Latest reference range and units	Day 1 of admission (morning value)	Day 1 of admission (evening value)	Day 2 of admission (morning value)	Day 2 of admission (evening value)	Day 3 of admission (morning value)	Day 3 of admission (evening value)	Day 4 of admission (morning value)	Day 4 of admission (afternoon value)	Day 4 of admission (evening value)	Day 5 of admission (morning value)	Day 5 of admission (evening value)	Day 6 of admission
Sodium	136-145 mmol/L	145	142	145	144	141	144	139	141	140	137	140	139
														Potassium	3.5-5.1 mmol/L	1.8	2.6	2.1	1.9	2.6	2.4	3.7	2.9	3.9	4.3	4.0	4.0

**Table 2:** CPK trend during the course of admission CPK - creatine phosphokinase

	Latest reference range and units	Day 1 of admission	Day 2 of admission	Day 3 of admission	Day 4 of admission	Day 5 of admission	Day 6 of admission
CPK	20-170 U/L	9,306	9,564	12,057	>22,000	20,282	7,522

**Figure 3:**
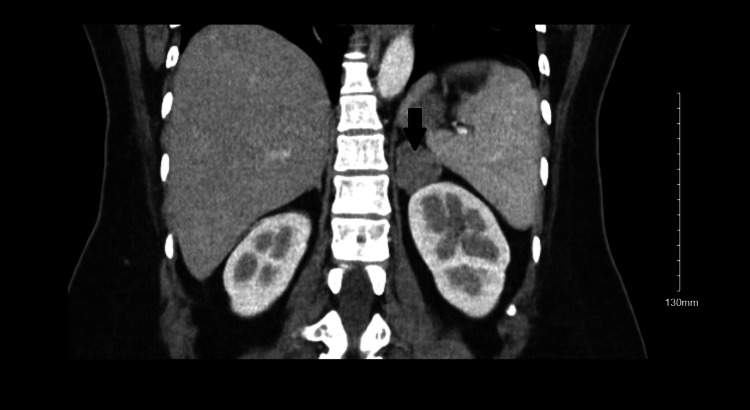
CT abdomen pelvis (coronal view) showing left-sided adrenal adenoma

**Figure 4:**
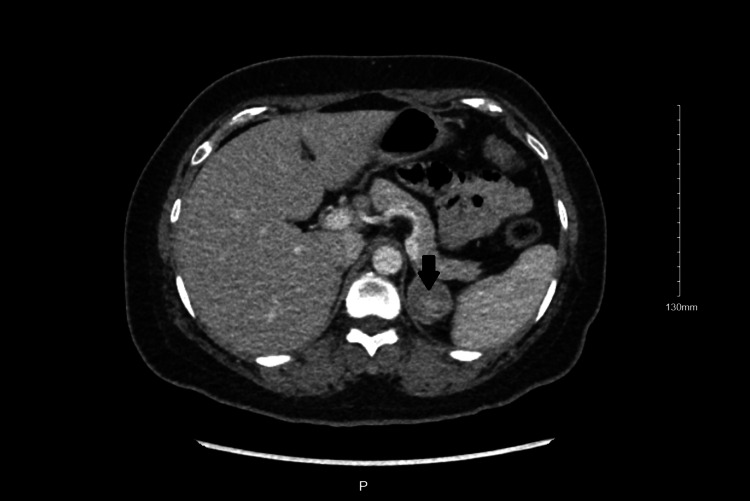
CT abdomen pelvis (transverse view) showing left-sided adrenal adenoma

A month later, she had a dedicated CT abdomen with contrast, which showed a 3.2 cm left adrenal nodule with precontrast, dynamic, and delayed phase imaging as 16, 81, and 36 Hounsfield units, respectively. The relative percent washout value was greater than 40% and was compatible with adenoma (Figure 3 and Figure 4). The right adrenal gland was unremarkable. Her latest potassium level was 4.8 mmol/L with potassium supplementation and spironolactone. Laparoscopic adrenalectomy was then performed, after which her potassium and aldosterone levels returned to normal. Histopathology report further supported the diagnosis of an adrenal adenoma due to positive melanin A and calretinin, focal weak positive synaptophysin, and negative chromogranin and paired-box gene 8 (PAX8). After her discharge, she had no recurrence of her symptoms.

## Discussion

Rhabdomyolysis is a pathological condition in which skeletal muscle breakdown leads to the release of muscle-breakdown products such as CK, myoglobin, lactic acid, and lactate dehydrogenase (LDH) into the bloodstream [2]. The causes of rhabdomyolysis are typically categorized as physical and non-physical. Physical causes include crush syndrome in approximately 37% of cases, intense physical activity, e.g., strength training, which makes up about 38% of cases related to physical activity, and muscle exertion due to seizures [4]. Non-physical causes include multiple factors such as chronic alcoholism, drugs like cocaine and amphetamines, medications like statins, infections, inflammatory myopathies, heat stroke, neuroleptic malignant syndrome (NMS), and genetic disorders of glycogen storage like McArdle's disease [2]. During pregnancy, women are at risk of hypokalemia-induced rhabdomyolysis due to causes such as hyperemesis-induced hypokalemia and underlying distal renal tubular acidosis, which can be unmasked in pregnancy [5,6]. There has also been a reported case of rhabdomyolysis following intravenous iron sucrose administration during pregnancy [7]. Meanwhile, in non-pregnant adults, iron-dextran and ferric gluconate supplements have led to rhabdomyolysis [8,9]. In this case, the patient had no history of anemia or iron supplementation during or after her pregnancy. She reported using a percussion massage gun to alleviate cramps and muscle weakness, the use of which has been attributed as a cause of rhabdomyolysis [3]. However, the discovery of an adrenal adenoma and the subsequent resolution of her symptoms suggest that the adrenal pathology was most likely the causative factor. 

Rhabdomyolysis, the breakdown of muscle tissue, releases potassium into the bloodstream, leading to hyperkalemia; it also releases toxic substances like myoglobin, which can result in multi-organ failure, with AKI being the most serious complication. Increased levels of myoglobin can cause up to 40% of patients with rhabdomyolysis to develop acute renal failure [10]. A commonly used clinical definition for rhabdomyolysis is CK levels almost five times the normal limit, and typically, CK levels >5,000 are correlated with severe muscle injury and AKI [2]. This patient was diagnosed as having rhabdomyolysis due to extremely high CK levels (9,306 U/L) on presentation, which further rose to a maximum of 22,000 U/L.

Rhabdomyolysis associated with hypokalemia is a rare presentation, as hyperkalemia is generally the expected outcome of muscle breakdown. Typically, rhabdomyolysis occurs when potassium levels fall below 2.0 mmol/L [11]. Hypokalemia can be due to several causes; these include medications such as diuretics and laxatives, diarrhea, renal diseases like types I and II renal tubular acidosis, inadequate nutrition, and insulin overdose [12]. Low potassium levels lead to ECG findings like T-wave and ST segment depression, QT-prolongation, and the appearance of a U-wave - potentially causing fatal cardiac arrhythmias [13]. Other complications may include intestinal and respiratory paralysis [14]. Hypokalemia usually presents as progressive muscle weakness, more commonly in the lower limbs, as seen in this patient, along with other symptoms such as fatigue, constipation, and palpitations [13].

Another cause of hypokalemia is hormonal imbalance, particularly that of aldosterone. This can result from primary hyperaldosteronism, also known as Conn Syndrome, which may be caused by either an adrenal adenoma (30%) or BAH (60%) [15]. Secondary hyperaldosteronism is usually due to hyperactivity of the renin-angiotensin-aldosterone system (RAAS), usually due to a renin-producing tumor. Excessive aldosterone causes sodium retention and potassium excretion due to the activation of mineralocorticoid receptors on renal cells, which then leads to sodium reabsorption via the epithelial sodium channels (ENaC) on the luminal side of the cortical collecting duct and potassium excretion via the sodium-potassium exchange pumps. Sodium reabsorption leads to water retention, which, alongside aldosterone's direct action of vasoconstriction, causes hypertension [16]. In the majority of cases, potassium levels are within the normal range. Because hypokalemia is observed in only about 28.1% of patients with hyperaldosteronism, this case report highlights how unusual it is for hypokalemia to be severe enough to result in rhabdomyolysis [17].

One proposed explanation for the connection between hypokalemia and rhabdomyolysis is that potassium plays a role in dilating arterioles in skeletal muscles, particularly during physical activity. This dilation is impaired when serum potassium levels are low, resulting in reduced blood flow and relative ischemia in the muscle fibers [18]. Another theory suggests that hypokalemia inhibits the production and storage of glycogen within cells and disrupts normal ion movement across cell membranes [19]. Given the vital role potassium plays in maintaining normal physiological functions like muscle contraction and normal heart rhythm, inpatient management of such patients is crucial to replete total body potassium stores and correct serum potassium levels to prevent them from developing critical illnesses such as muti-organ failure and AKI.

## Conclusions

Primary hyperaldosteronism leading to hypokalemia is a rare but important cause of rhabdomyolysis and should be considered as a differential when patients present with muscle weakness and lab findings consistent with rhabdomyolysis. Therefore, physicians must maintain a high degree of clinical suspicion for this diagnosis, especially in the setting of muscle weakness, elevated CK levels, and low serum potassium. Recognizing the potential link between hypokalemia and rhabdomyolysis is essential for guiding further investigation and ensuring appropriate management of affected patients. Early recognition and intervention can prevent serious complications, ultimately improving patient outcomes.
